# The anti-osteosarcoma effect from panax notoginseng saponins by inhibiting the G_0_ / G_1_ phase in the cell cycle and affecting p53-mediated autophagy and mitochondrial apoptosis

**DOI:** 10.7150/jca.54602

**Published:** 2021-08-30

**Authors:** Guangtao Han, Yubiao Zhang, Ting Liu, Jianping Li, Haohuan Li

**Affiliations:** 1 Department of Orthopedics, Renmin Hospital of Wuhan University, Wuhan, Hubei 430060, P.R. China; 2 Department of Orthopedics, Hospital of Shenmu, Shenmu, Shaanxi, 719300, P.R. China

**Keywords:** apoptosis, autophagy, panax notoginseng saponins, osteosarcoma, p53, G_0_ / G_1_ phase

## Abstract

Osteosarcoma is the most common primary bone malignancy, and current chemotherapy sessions against it often induce severe complications in patients. Thus, it is necessary to develop new and effective antineoplastic agents with fewer side effects. Panax notoginseng saponins (PNS) are the active components in panax notoginseng and were reported to be capable of inhibiting the growth of several tumors both in vitro and in vivo. However, its effects on osteosarcoma have not been studied yet, which is addressed in this study for the first time. Our results indicated that PNS can inhibit proliferation, invasion and migration of the osteosarcoma cells, while promoting their apoptosis simultaneously. Specifically, PNS caused an increase in mitochondrial membrane potential and the amount of reactive oxygen species. The cell cycle in osteosarcoma cells was arrested in the G0 / G1 phase after PNS treatment. The expression of p53 and other apoptosis-related mitochondrial proteins were promoted. Nevertheless, it was observed that autophagy became less active in osteosarcoma cells when PNS was administered. In a word, PNS were of potential therapeutic significance for osteosarcoma.

## Introduction

Osteosarcoma is a rare malignancy that occurs in children and adolescents, which happens at a rate of about 10 in a million 1. It is highly malignant and tends to metastasize at the early stage 2. For patients with osteosarcoma, most current treatments depend on neoadjuvant chemotherapy followed by surgical resection 3. However, chemotherapy often introduces severe complications in the patients 4. Thus, it is necessary to develop new effective antineoplastic agents with fewer side effects.

Autophagy is a process in which aged or damaged cells were surrounded by autophagosomes, which fused with lysosomes to form autosomes for debris degradation 5,6. This process can prevent accumulation of damaged cell debris and help keep homeostasis in the micro-environment 7. On the other hand, apoptosis is the classical mechanism of induced cell death 8. Generally, autophagy is considered to be a protective response to stress and counteracts against on apoptosis; in other words, autophagy inhibits apoptosis 9. A good balance between the two will be beneficial to control tumor development.

Panax notoginseng saponins (PNS) are the extraction products from panax notoginseng, whose pharmaceutical effects have been widely studied in recent years 10-15. According to the published literature, PNS can facilitate both autophagy and apoptosis in several tumors 16, 17. However, different cells may exhibit different biological features during identical chemical treatments. As PNS have been reported to regulate autophagy and apoptosis in some other cells, in this study, potential influence of PNS on osteosarcoma growth, as well as the mechanisms behind such influence with regards to apoptosis and autophagy was investigated. It was found that PNS can inhibit osteosarcoma cell proliferation and migration, increase mitochondrial membrane potential and control osteosarcoma in the G_0_ / G_1_ phase. Unlike the results obtained from other tumors, PNS were found to enhance mitochondrial apoptosis but inhibit autophagy in osteosarcoma cells.

## Materials and methods

### Antibodies and reagents

DMEM/F12 high glucose was provided by Hyclone (Utah, USA). Fatal bovine serum(FBS), trypsin, penicillin-streptomycin, Annix-V/PI kit, Cell Counting Kit-8(CCK-8), JC-1 kit and ROS kit were obtained from Gibco (New York, USA). Transwell chambers with membranes at a pore size of 8 μm were bought from Corning Life Science (New York, USA). Hoechst 33258 was purchased from Sigma-Aldrich (St Louis, MO, USA). PI was purchased from MultiSciences (Zhejiang Province, China). Primary antibodies against p53, p27, cleaved caspase-9, cleaved caspase-3, Bcl-2, Bax, cyclinD1, cyclin-dependent kinase 2 (CDK2), cytochrome c, apoptotic protease activating factor 1 (Apaf-1), LC3-I, LC3-II and glyceraldehyde 3-phosphate dehydrogenase (GAPDH) were provided by Cell Signaling Technology (Beverly, MA, USA). Horseradish peroxidase (HRP)-conjugated secondary antibodies were provided by Huabio (Hangzhou, Zhejiang, China). Pifithrin-α (PFT-α) was provided by Selleck Chemicals (Shanghai, China). PNS was provided by Kunming Pharmaceutical Company (Kunming, China).

### Cell culture

Osteosarcoma 143B and HOS cells were obtained from China Centre for Type Culture Collection (Wuhan, China). The Cells were cultured in DMEM/F12 medium containing 1% penicillin/streptomycin and 10% FBS in a conventional incubator at 37 °C with 5% CO_2_.

### Cell viability assay

Cell viability was measured by a CCK-8 kit following the manufacturer's instructions. Briefly, in a nutshell, cells were seeded in a 96-well plate at a concentration of 5×10^3^ cells/well. After 24 hours of incubation, the medium was replaced by several new media containing PNS at a concentration of 0, 100, 200, 300 μg /ml, respectively. The wells were then separated into two groups, followed by another 24 hours or 48 hours of incubation. Subsequently, 10μL of CCK-8 solution was added to each well, and cell viability was measured at 450 nm with a micro tablet reader (Tecan Sunrise, Salzburg, Austria) 1.5 hours later.

### Cell apoptosis determination

Cells were collected for apoptosis determination by an AnnixV-fluorescent isothiocyanate (FITC)/sodium propanodide (PI) cell apoptosis detection kit. In short, cells were resuspended in 1×binding buffer, and 5 μL FITC and 5 μL PI solutions were added. After incubation at 37 °C for 15 minutes, cell apoptosis were analyzed by flow cytometry (Becton-Dickinson, New Jersey, USA).

### Wound-healing assay

Here, 10^6^ cells were seeded into a six-well plate and allowed for fusion. The osteosarcoma cell monolayer was mechanically scratched with a 200 μL sterile pipette tip. After washed twice with phosphate-buffered saline (PBS), cells in DMEM/F12 medium (without FBS) were treated with different concentrations of PNS, and then cultured for a certain period of time. A series of images were captured with a microscope (Olympus IX51) to observe cell migration 0 and 24 hours after scratching.

### Colony-formation assay

Placed in a six-well plate at a density of 5×10^2^ cells/well, osteosarcoma cells were treated by different concentrations of PNS in the treatment groups and incubated for another 2 weeks along with the nc group, where no PNS were administered. Methanol was used to fix the colonies that were stained with the Wright-Giemsa solution. After the 2-week incubation, all plates were counted for the number of colonies.

### Nuclear staining analysis by Hoechst 33258

After 24 hours of PNS treatment, osteosarcoma cells were washed twice with PBS and incubated with Hoechst 33258 at room temperature in the dark for 15 minutes. Subsequently, the cells were rinsed with PBS twice, and their photographs were taken by an inverted fluorescent microscope (Olympus BX51; Olympus, Tokyo, Japan) (×200), where apoptotic cells showed a bright-blue fluorescence.

### Measurements of mitochondrial membrane potential (MMP)

JC-1 was performed to evaluate mitochondrial membrane potential. 5×10^5^ cells were rinsed with PBS and trypsinized at 37 °C for 2 min, followed by incubation with 0.5 ml of JC-1 staining working solution at 37 °C for 20 min. After stained with JC-1, MMP was measured by flow cytometry.

### Measurements of mitochondrial permeability transition pore (MPTP)

Osteosarcoma cells were seeded at a concentration of 5×10^5^ cells/ml, After that, 3 μl of working solution and 5 μl of fluorescence quencher were added to each sample, then the samples were placed in a dark room at 37 °C for 15 min. Next, after washed with 3 ml of MPTP buffer, the cells were resuspended with 400 ml of Hanks' balanced salt solution (HBSS). The alterations in fluorescence intensity for each sample were measured by flow cytometry, and the decrease in relative fluorescence units was used to evaluate the open extent of MPTP.

### Analysis on reactive oxygen species (ROS)

Following the manufacturer's instructions, cells at a concentration of 5×10^5^ cells/ml were detected by dichlorodihydrofluorescein diacetate (DCHF DA). Briefly, 10 μl of DCHF DA was stained in the cell-containing medium at room temperature for 2 hours. After staining, cells were collected and washed twice with pre-chilled PBS, and the ROS amount within was determined by flow cytometry.

### Transwell assays

Transwell assays were used to evaluate cell migration. Chambers were first washed with serum-free medium, and then three sets of 600 μl of treatment medium, with 10% FBS plus no PNS, 100 μg/ml or 200 μg/ml of PNS, were added to the lower chamber. 200 µl of cell solution containing 10^6^ cells/ml was loaded in the upper chamber. After 8 hours of incubation, the chambers were fixed with 4% paraformaldehyde for 15-20 min and stained with crystal violet for 15 min afterwards. Finally, the cells were wiped off from the upper membrane, and those on the lower membrane were counted with an inverted microscopy.

### Analysis on the cell cycle

Firstly, osteosarcoma cells at a concentration of 2-3×10^5^ cells per well were planted into a 6-well plate, added with PNS of different concentrations (0, 100 μg/ml, 200 μg/ml) and incubated at 37 °C for 24 hours. Then, these cells were washed with PBS and collected, followed by immobilization in 75% cold ethanol and storage at -20 °C for at least 2 hours. Subsequently, the cells were centrifuged at 12000 bpm for 5 min and hydrated with PBS for 15 min, and the supernatant was aspirated afterwards. Finally, the cells were centrifuged again at a speed of 12000 bpm for 5 min and stained with 50 μg/ml of PI (MultiSciences, Zhejiang Province) for 30 min for flow cytometry analysis.

### Western blot

Expressions of p27, cyclin D1, CDK2, Bcl-2, P53, Bax, Cytochrome c, cleaved caspase 9, cleaved caspase 3, Apaf-1, LC3-I and LC3-II proteins in osteosarcoma 143B and HOS cells were analyzed by Western blot assays. After treated with different concentrations of PNS, osteosarcoma cells were centrifuged and washed twice with PBS. After that, they were lysed and centrifuged at 4 °C for 12 min at 12000 r/min to collect total protein, and a certain amount of protein loading buffer was added and boiled at 100 °C for 15 min. Following that, a BCA protein assay kit was used to calculate the protein amount. Briefly, the proteins were denatured for polyacrylamide gel electrophoresis (12%, SDS-PAGE), followed by a transfer onto a 0.45 μm polyvinylidene fluoride membrane (PVDF). The PVDF membrane was blocked with 5% skimmed milk powder for 1 h at room temperature, and primary antibody was added onto the membrane, which was incubated overnight. Subsequently, the PVDF membrane was washed with TBST buffer (TRIS-HCL balanced salt buffer + Tween) for 3 times, and incubated with a secondary antibody at room temperature in the dark for 1 h. Then the bands were twice with TBST buffer and scanned by the Odyssey infrared laser imaging system. Specifically, the optical density of the target band was obtained and analyzed by the gel image processing system.

### Statistical analyses

SPSS 20.0 statistical software package (SPSS Inc, Chicago, Illinois) was used to process the results. The experimental results were expressed as mean ± standard deviation (SD), and all experiments were repeated in triplicates. One-way analysis of variance was implemented and least significant difference (LSD) numbers were obtained. Specifically, P<0.05 was defined as an indicator for significant difference.

### Ethical statements

All data in this paper was obtained from cell lines. No patients or animals were directly involved or intervened to retrieve the experimental data. Therefore, ethical approvals were not necessary.

## Results

### PNS inhibit the proliferation of osteosarcoma cells

**Figure 1:**
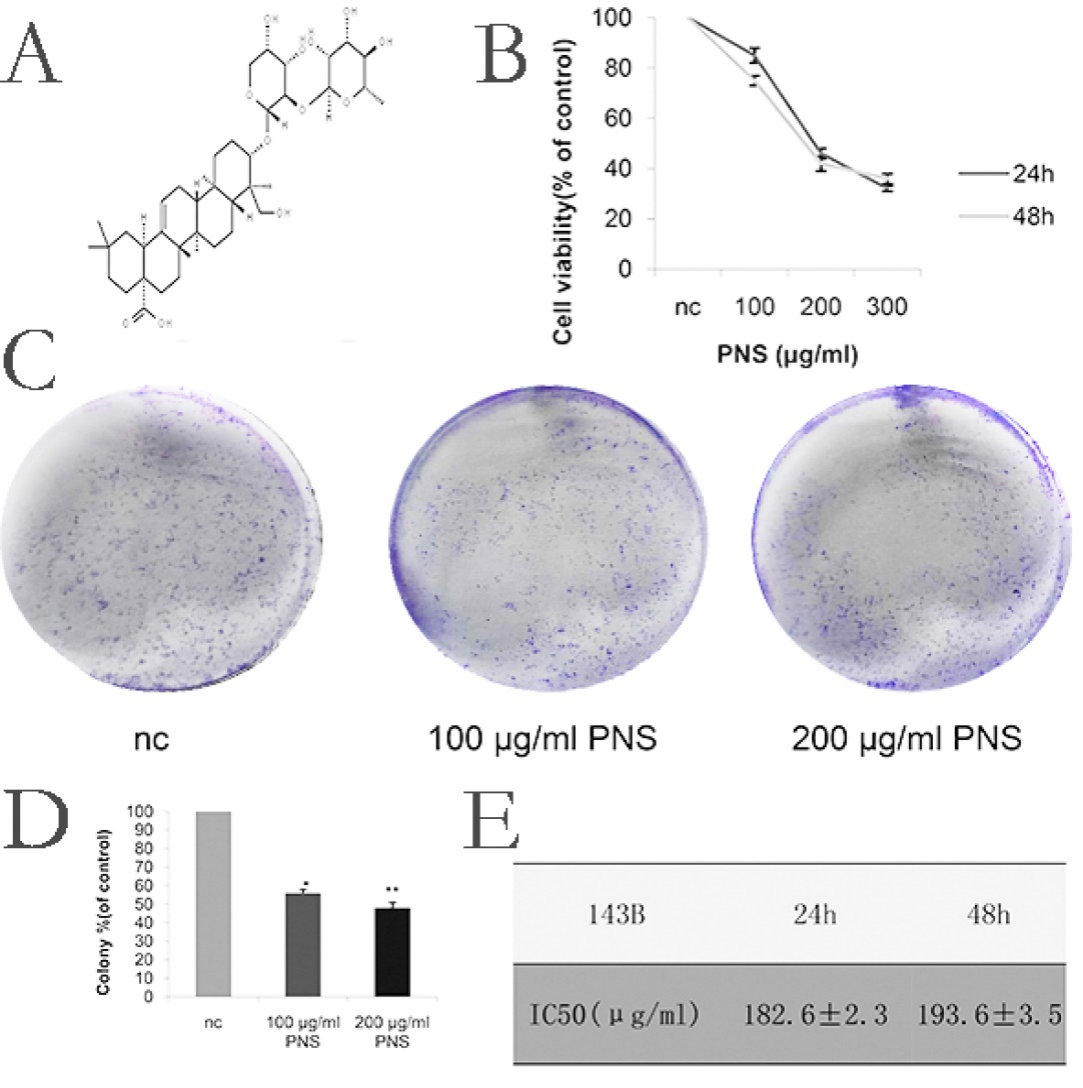
PNS cause a rise in the cytotoxicity of osteosarcoma 143B cells and inhibit their proliferation in vitro. A. The chemical structure of PNS. B. CCK-8 test results on the cell viability of PNS-treated osteosarcoma 143B cells after 24h and 48h. C-D. Plate cloning assay results and data analysis. The osteosarcoma cells were incubated with different concentrations of PNS (0, 100 μg/ml & 200 μg/ml) for 24 hours, and placed in the medium for 14 days. E. Analysis of IC_50_ of PNS in osteosarcoma 143B cells. Data are expressed as mean ± standard deviation. * means P <0.05 vs nc, ** means P <0.01 vs nc. All experiments were performed in triplicates.

Firstly, in order to determine the appropriate concentration for PNS (whose chemical structure is shown in Figure 1A) to act on osteosarcoma, CCK-8 tests were carried out for PNS at different concentrations. As is shown in Figure 1B, 1E, 6A and 6B, the optimal PNS concentrations in osteosarcoma 143B cells at 24 h and 48 h were (182.6±2.3) μg/ml and (193.6±3.5) μg/ml respectively; in HOS cells, these concentrations became (293.8±3.5) μg/ml and (299.1±5.2) μg/ml, respectively. Furthermore, the 143B cells were divided further into three groups, namely nc (0μg/ml), low concentration (100μg/ml) and normal concentration (200μg/ml) groups. For HOS cells, three identical groups were separated as well, with PNS concentrations of 0 μg/ml, 150 μg/ml and 300 μg/ml assigned to nc, low concentration and normal concentration groups, respectively. To conclude, the proliferation of osteosarcoma cells was found to be negatively correlated with PNS concentration, as is shown in Figure 1C & 1D.

### PNS promotes the apoptosis of osteosarcoma cells

**Figure 2:**
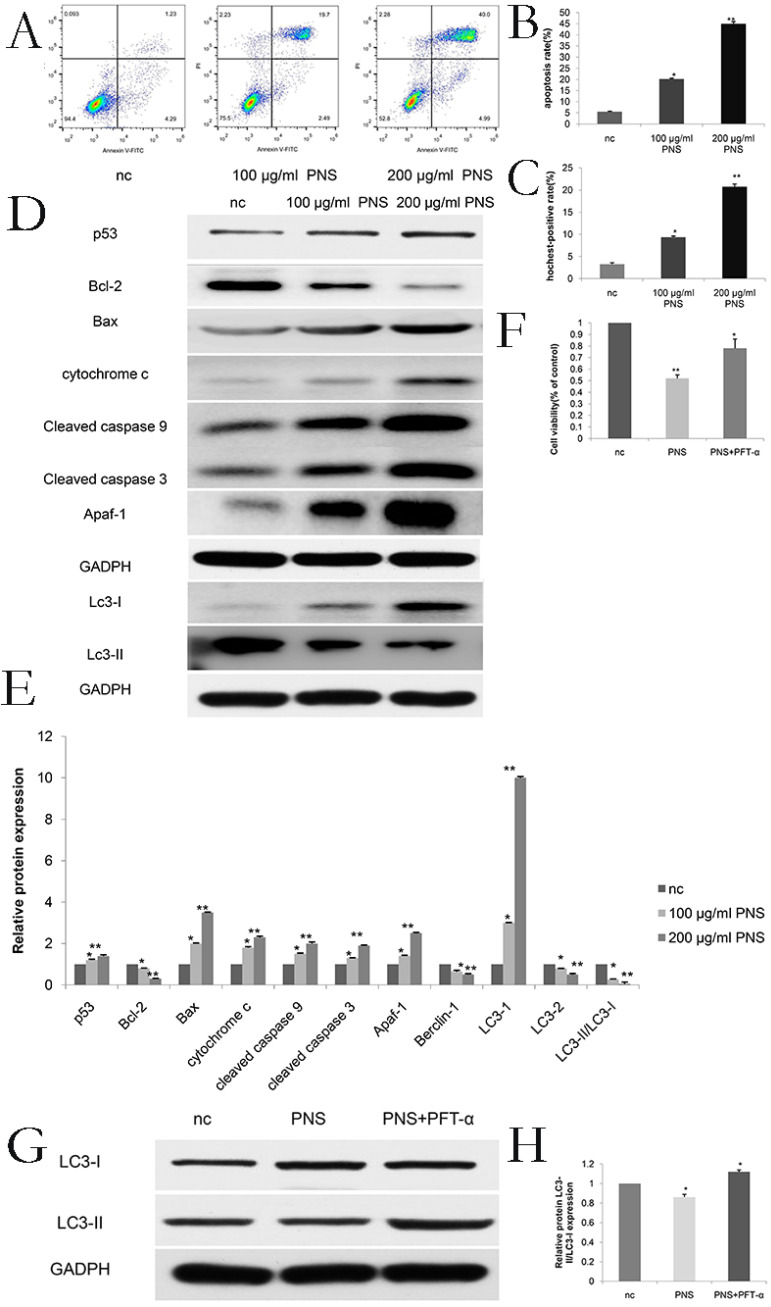
PNS promote the apoptosis of osteosarcoma 143B cells in vitro. A-B. Annix V / PI double staining assays were used to detect and analyze the apoptosis extent of osteosarcoma 143B cells. PNS concentration was positively correlated to the apoptosis rate. C. Hochest 32258 analyzed the morphology of osteosarcoma cells quantitatively. Bright blue fluorescent nuclei represented apoptotic osteosarcoma cells. D-F. Western Blot was used to detect and analyze the expressions of p53 and other apoptosis-related or autophagy-related proteins. Before adding PNS into wells with osteosarcoma 143B cells, PTF-α was added, and the cell proliferation rate was analyzed by CCK-8. G-H. Western Blot was used to detect and analyze autophagy-related proteins. Before adding PNS into wells with osteosarcoma 143B cells, PTF-α was added. Data are expressed as mean ± standard deviation. * means P <0.05 vs nc, ** means P <0.01 vs nc. All experiments were performed in triplicates.

AnnixV-FITC/PI experiments indicated that with higher PNS concentrations, their effects on apoptosis of osteosarcoma cells became more obvious. Particuarly, the difference in apoptotic effect between the 100 μg/ml and nc groups and that between the 200 μg/ml and nc groups were both statistically significant (P <0.05) (Figure 2A & 2B). Hochest 32258 was used to display the apoptosis of osteosarcoma cells. In Figure 2C, bright blue spots indicate apoptotic cells, and it can be observed that PNS could significantly promote apoptosis in a concentration-dependent way. In addition, PNS could lead to an increase in the expression of p53, cytochrome c, cleaved caspase 9, cleaved caspase 3, Apaf-1 and Bax proteins, but could result in a drop in the expression of Bcl-2. Such effects were demonstrated by WB to be dose-dependent (Figure 2D, 2E, 6C & 6D). To further verify such findings, PFT-α (a p53 inhibitor) was used to detect the extent of apoptosis in PNS-treated osteosarcoma cells. The results showed that 30 μM of PFT-α could effectively inhibit osteosarcoma cell apoptosis (Figure 2F, 6G). Therefore, it suggested that PNS could promote osteosarcoma cell apoptosis by activating the p53 mitochondrial pathway.

### PNS affect the expression of p53 and autophagy related genes in osteosarcoma cells

To investigate the effect of PNS on p53 and autophagy, expression of LC3-I and LC3-II proteins were studied. The results showed that PNS caused p53 expression to rise, while the proportion of LC3-II/LC3-I reduced. Such effect was found to be dependent on PNS concentration (Figure 2D, 2E, 6E & 6F). To further confirm such results, PFT-α (a p53 inhibitor) was used to detect the autophagy extent of osteosarcoma cells. The results showed that 30 μM of PFT-α could effectively promote autophagy in these cells (Figure 2G, 2H, 6H, 6I), indicating that PNS could affect the expression of autophagy related genes by the p53 pathway.

### PNS inhibit the migration and invasion of osteosarcoma cells

**Figure 3:**
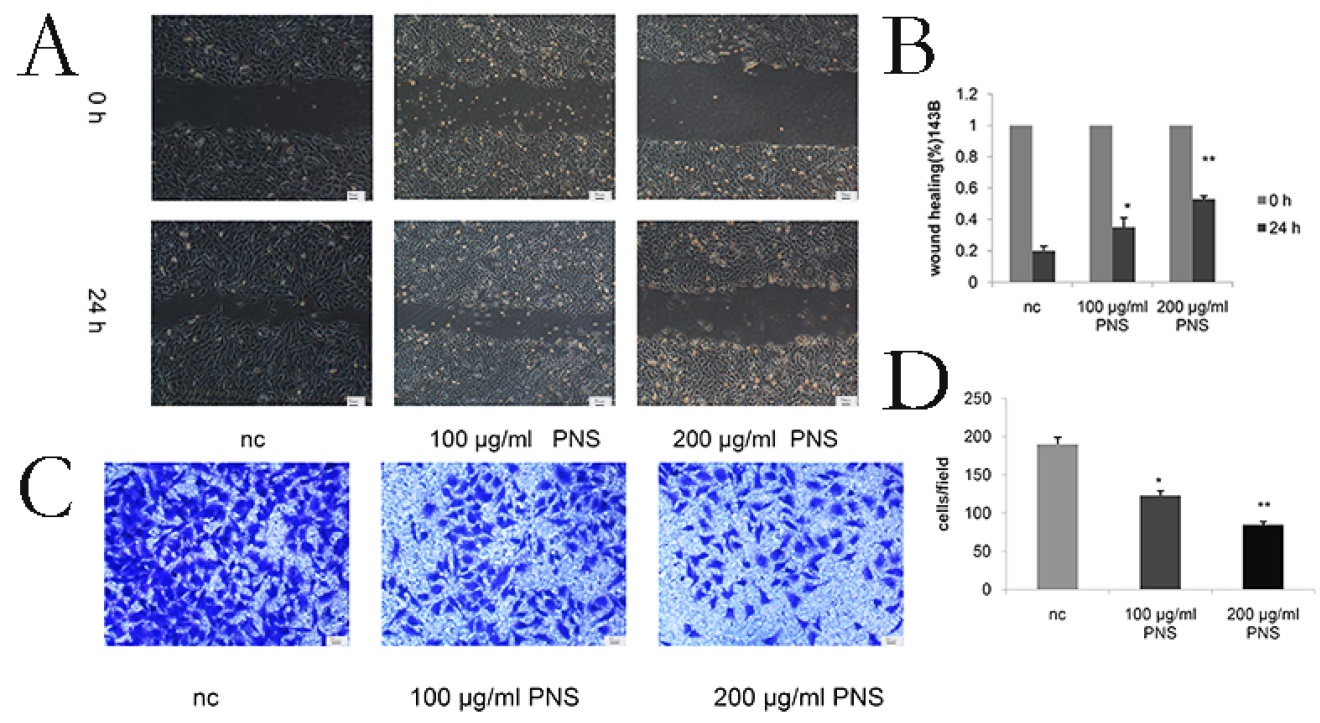
PNS inhibit the invasion and migration of osteosarcoma 143B cells in vitro. A-B. The wound healing experimentwas used to quantitatively analyze the migration of osteosarcoma cells. C-D. Transwell assays were utilized to quantitatively determine the effects of PNS on osteosarcoma invasion. Data are expressed as mean ± standard deviation. * means P <0.05 vs nc,** means P <0.01 vs nc. All experiments were performed in triplicates.

Wound healing assays were conducted to detect the migration of osteosarcoma cells. It was found that PNS concentration was negatively correlated with the migration rate of osteosarcoma cells (Figure 3A & 3B). The difference between the 100 μg/ml and nc groups, as well as that between the 200 μg/ml and nc groups, was statistically significant (P<0.05). Cell invasiveness was measured by transwell assays, and PNS were discovered to be able to significantly inhibit osteosarcoma cell invasion (Figure 3C & 3D).

### PNS affect the G_0_/G_1_ phase in osteosarcoma cells

**Figure 4:**
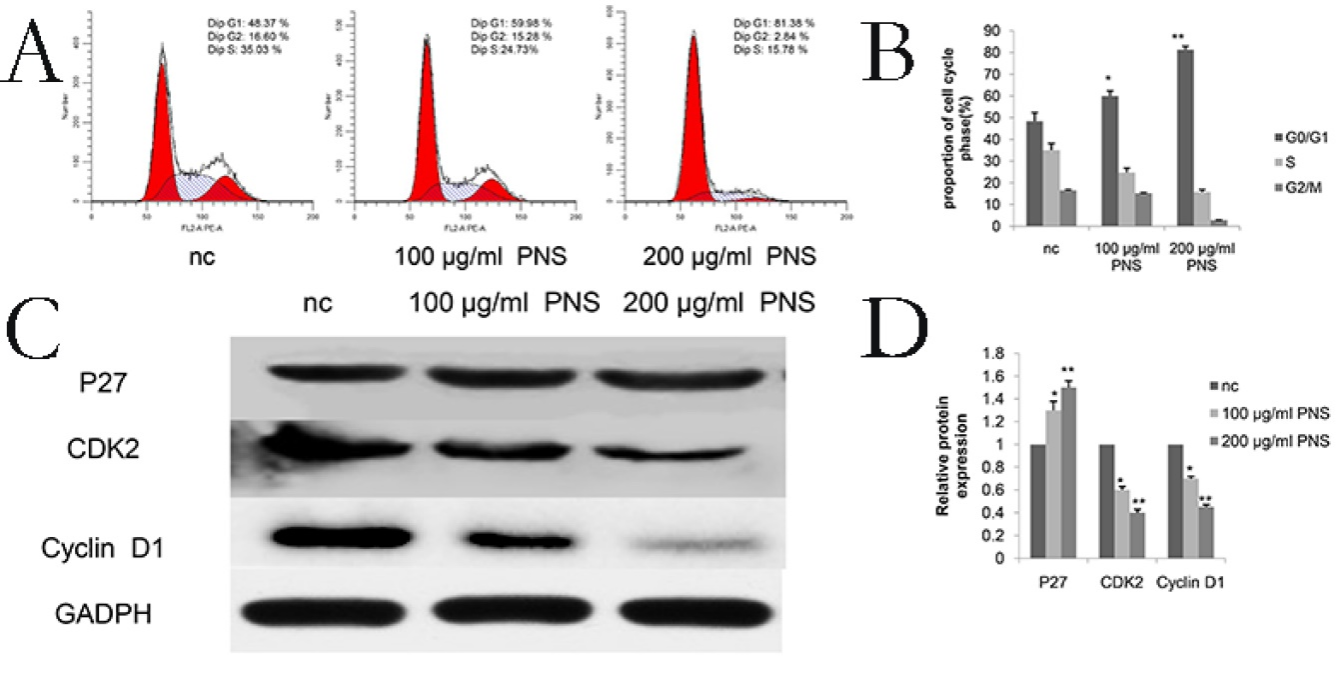
PNS confine osteosarcoma 143B cells in the G0 / G1 phase in vitro. A-B. Osteosarcoma cells were treated with PNS for 24 hours, and flow cytometry was used to quantify the DNA content at each stage in the cell cycle. The results indicated that PNS constrain osteosarcoma 143B cells in the G_0_ / G_1_ phase. C-D, Western blot was used to quantitatively analyze the effects of PNS on proteins related to the cell cycle. Data are expressed as mean ± standard deviation. * means P <0.05 vs nc, ** means P <0.01 vs nc. All experiments were performed in triplicates.

Cell cycle assay results showed that PNS obviously influenced the G_0_/G_1_ phase in osteosarcoma cells (Figure 4A & 4B). Specifically, proteins involved in the cell cycle were studied, and it was found that PNS would lead to a reduction in the expression of cyclinD1 and CDK2, but P27 showed an increase in its amount (Figure 4C, 4D, 6E & 6F).

### PNS increase the ROS level, decrease mitochondrial membrane potential and promote the opening of MPTP on osteosarcoma

**Figure 5:**
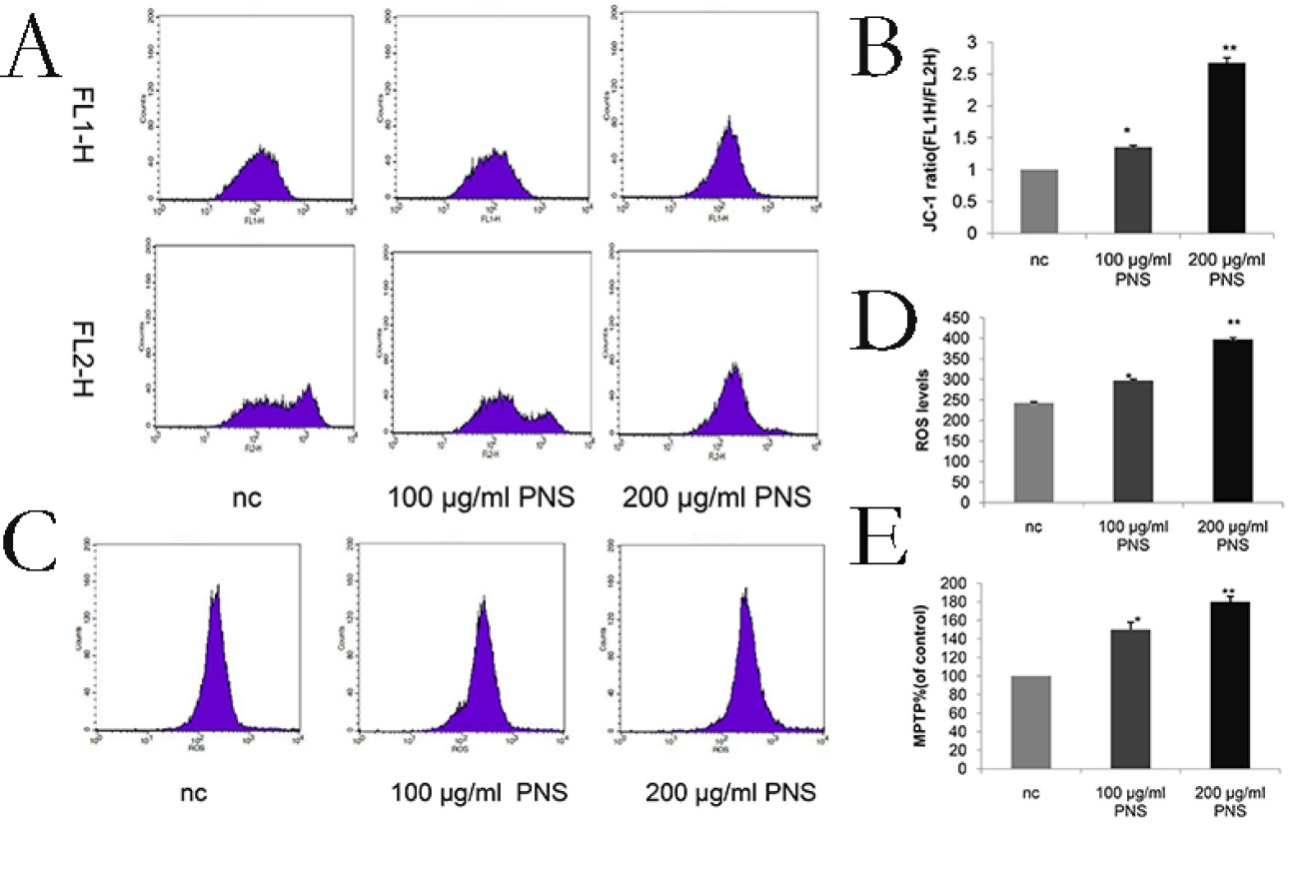
Effect of PNS on mitochondrial membrane potential and reactive oxygen amounts in osteosarcoma 143B cells. A-B. PNS effects and the quantitative analysis on mitochondrial membrane potential of osteosarcoma cells. F1L-H stands for apoptotic cells, and F2L-H represents normal cells. C-D. PNS effects and the quantitative analysis on reactive oxygen level in osteosarcoma cells. Data are expressed as mean ± standard deviation. E. MPTP of PNS-treated osteosarcoma 143B cells.* means P <0.05 vs nc, ** means P <0.01 vs nc. All experiments were performed in triplicates.

**Figure 6:**
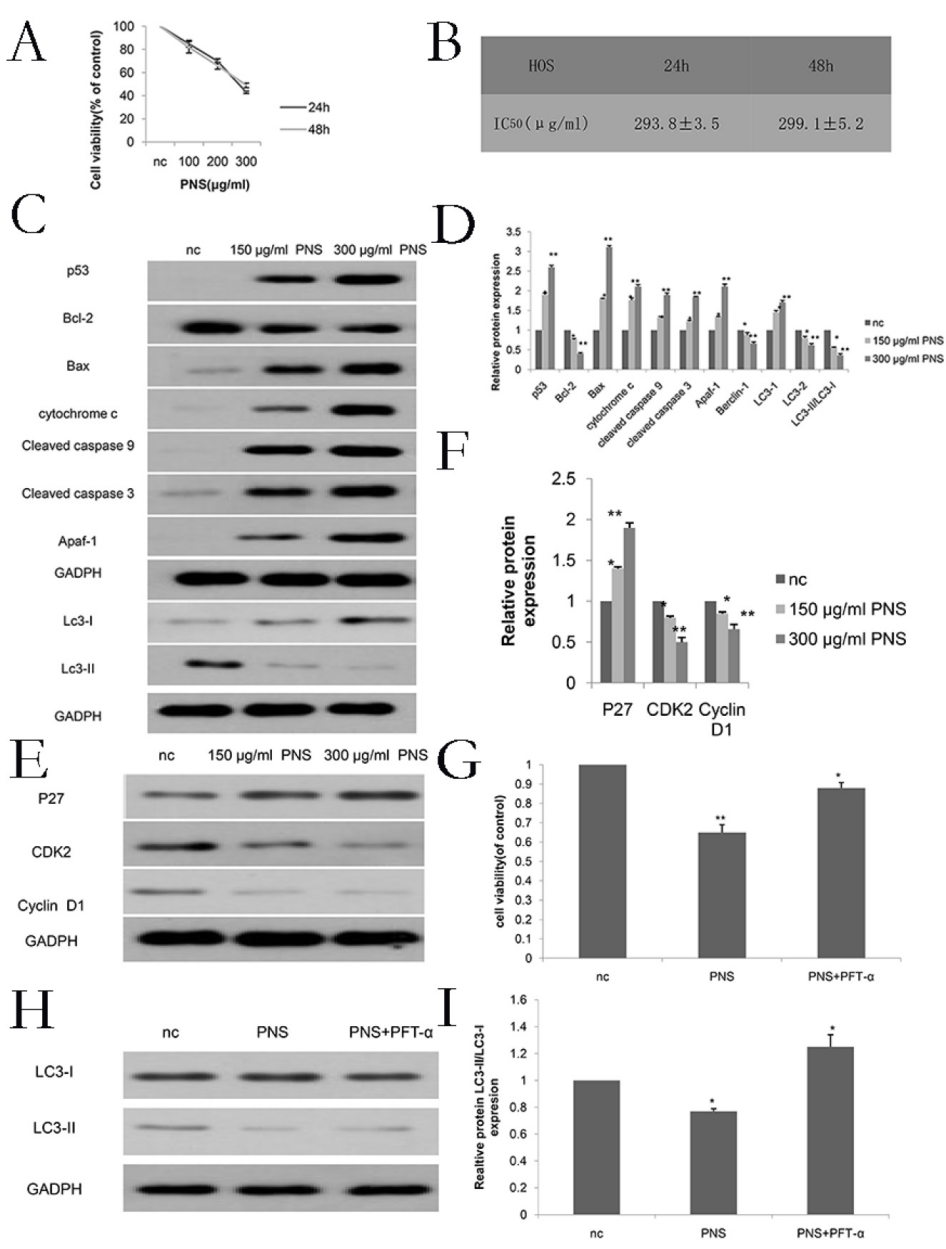
PNS were observed to contribute to the increase in the cytotoxicity of osteosarcoma HOS cells. Besides, they were also found to promote osteosarcoma apoptosis and constrain osteosarcoma cells in the G_0_ / G_1_ phase in vitro. A-B. CCK-8 assays were used to determine the cell viability of PNS-treated osteosarcoma HOS cells after 24 h and 48 h. C-F. Western Blot assays were used to detect and analyze the expression of p53, as well as other proteins related to apoptosis, cell cycle and autophagy. G-I, Before treating osteosarcoma cells with PNS, PTF-α was used to treat the cells. The expression of autophagy-related genes was analyzed by Western Blot, and the cell proliferation rate was analyzed by CCK8.* means P <0.05 vs nc, ** means P <0.01 vs nc. All experiments were performed in triplicates.

ROS levels were a sign of early apoptosis. An increase in the ROS level will result in the loss of mitochondrial membrane potential and alterations in the size of MPTP. After a 24-hour PNS treatment, flow cytometry was performed to measure the ROS level and found a significant increase (Figure 5C & 5D). In addition, MMP and MPTP were used to verify the effect of PNS on osteosarcoma cells. It was observed that PNS obviously promoted the opening of MPTP and facilitated lower mitochondrial membrane potential in a dose-dependent manner (Figure 5A, 5B & 5E).

## Discussion

Compounds from medicinal plants may be of pharmacological values. Specifically, PNS play an important role in clinics to treat myocardial ischemia, cerebral infarction, atherosclerosis and several other metabolic diseases 18-21. Recently, PNS have been reported to exhibit anti-tumor activities. P Wang et al. pointed out that PNS can inhibit the metastasis of breast cancer 22. ZG Yang et al. proved that PNS are helpful to treat cervical cancer by inducing apoptosis in Hela cells 23. CZ Wang demonstrated that PNS could inhibit the proliferation of colon cancer cells 24. Q Yang et al. concluded that PNS could slow down cell proliferation in lung cancer 25. Additionally, some early researches suggested that PNS can inhibit tumor cell proliferation as an inducer for both cell apoptosis and autophagy 16, 17. However, chemicals may take different functions in different cells, and the therapeutic effects of PNS on osteosarcoma have not been studied in previous literature.

In this study, PNS is reported for the first time to be capable of inhibiting the proliferation of osteosarcoma cells. Furthermore, the appropriate PNS concentration for treatment was determined by CCK-8 assays. The inhibitory effects from PNS were found to be both time-dependent and concentration-dependent. After PNS treatment, cell apoptosis was promoted. These results suggest that PNS may be of medical value for therapies against osteosarcoma.

Particularly, p53 is the guardian human gene, which is an important tumor suppressor 26. It is involved in several cellular processes, such as cell cycle control, apoptosis and autophagy 27-29. In these processes, it can induce cell cycle arrest and promote expression of apoptosis-related genes. Besides, it can affect the mitochondrial pathway by altering the expression of Bcl-2 and Bax proteins, leading to the loss of MMP, higher MPTP openness and mitochondrial dysfunction as a consequence. Following that, cytochrome C leaks into the cytoplasm, which activates the expression of Apaf-1, cleaved caspase 9 and cleaved caspase 3, resulting in the activation of apoptotic pathways. On the other hand, p53 can also inhibit autophagy by inhibiting LC3-II expression. In the present study, it is found that unlike other tumor cells in earlier researches, osteosarcoma cells showed a promoted apoptosis and inhibited autophagy after PNS treatment.

Cell metastasis is a sign of malignancy 30. Malignant metastasis of tumor cells will lead to treatment failure for osteosarcoma and a casualty rate of about 90%. However, in this study, PNS was revealed to effectively reduce the invasion and migration of osteosarcoma cells in a concentration-dependent manner.

A complete cell cycle is mainly composed of G_1_, S, G_2_ and M phases, which plays a role in cell growth and development 31. In the present study, a significant increase in the percentage of cells at the G_0_/G_1_ phase in osteosarcoma cell colonies was observed with PNS treatments, indicating that PNS inhibited osteosarcoma growth and development by inducing G0/G1 arrest. Specifically, CDK and Cyclin D proteins are synergistic to promote the formation of the G1 phase, and such effects are inhibited by p27, which works by binding to the D-CDK4 and E-CDK2 complexes. In this study, it was found that PNS could facilitate p27 expression while suppressing the expression of cyclin D1 and CDK2, so as to arrest osteosarcoma cells in the G_0_/G_1_ phase.

Increased apoptosis is often accompanied with proliferation inhibition in tumors 32, and osteosarcoma is of no exception. Based on the pathways involved, apoptosis can be categorized as death receptor-mediated or mitochondria-mediated. This study just focused on the effect of PNS on mitochondria-mediated apoptosis. In this study, MMP loss was verified as a higher permeability of the mitochondrial membrane and a rising amount of intracellular ROS as a result of PNS introduction. Furthermore, PNS was found to promote MPTP opening and up-regulate the expression of apoptosis-related proteins, which eventually facilitates mitochondrial apoptosis in osteosarcoma cells.

Mitochondrial functions, autophagy and apoptosis are all critical to maintain normal physiological functions 33, 34. Either excessive or too much inhibition on autophagy could induce apoptosis 35. In the present study, PNS treatment suppressed the autophagy in osteosarcoma cells, leading to a significant rise in the adaptability of these cells to the surrounding micro-environment. Such increase would cause damage in normal cells, such as abnormal metabolism or even death. On these aspects, our findings are consistent with previous researches about osteosarcoma 36-38.

In this study, it is demonstrated for the first time that PNS can cause G_0_/G_1_ arrest and promote apoptosis while inhibiting autophagy via the p53 pathway in osteosarcoma 143B and HOS cell lines. Although the effects of PNS on some other osteosarcoma cell lines, as well as those in vivo and the death receptor-mediate pathway, have not been addressed in this study, these findings still open the possibility of using PNS as a potential anti-tumor drug.

## Abbreviations

PNS: panax notoginseng saponins
FBS: fatal bovine serum
PI: propidium iodide
PBS: phosphate-buffered saline
FITC: fluorescent isothiocyanate
DCHF DA: dichlorodihydrofluorescein diacetate
PVDF: polyvinylidene fluoride membrane
TBST: TRIS-HCl balanced salt buffer + Tween
MMP: measurement of mitochondrial membrane potential
MPTP: mitochondrial permeability transition pore
ROS: reactive oxygen species
